# Identification and Characterization of Novel MicroRNAs from *Schistosoma japonicum*

**DOI:** 10.1371/journal.pone.0004034

**Published:** 2008-12-24

**Authors:** Xiangyang Xue, Jun Sun, Qingfeng Zhang, Zhangxun Wang, Yufu Huang, Weiqing Pan

**Affiliations:** 1 Institute for Infectious Diseases and Vaccine Development, Tongji University School of Medicine, Shanghai, China; 2 Department of Pathogenic Biology, Second Military Medical University, Shanghai, China; 3 Department of Microbiology, Wenzhou Medical College, University-town, Wenzhou, China; New England Biolabs, United States of America

## Abstract

**Background:**

Schistosomiasis japonica remains a major public health problem in China. Its pathogen, *Schistosoma japonicum* has a complex life cycle and a unique repertoire of genes expressed at different life cycle stages. Exploring schistosome gene regulation will yield the best prospects for new drug targets and vaccine candidates. MicroRNAs (miRNAs) are a highly conserved class of noncoding RNA that control many biological processes by sequence-specific inhibition of gene expression. Although a large number of miRNAs have been identified from plants to mammals, it remains no experimental proof whether schistosome exist miRNAs.

**Methodology and Results:**

We have identified novel miRNAs from *Schistosoma japonicum* by cloning and sequencing a small (18–26 nt) RNA cDNA library from the adult worms. Five novel miRNAs were identified from 227 cloned RNA sequences and verified by Northern blot. Alignments of the miRNAs with corresponding family members indicated that four of them belong to a metazoan miRNA family: let-7, miR-71, bantam and miR-125. The fifth potentially new (non conserved) miRNA appears to belong to a previously undescribed family in the genus Schistosome. The novel miRNAs were designated as sja-let-7, sja-miR-71, sja-bantam, sja-miR-125 and sja-miR-new1, respectively. Expression of sja-let-7, sja-miR-71 and sja-bantam were analyzed in six stages of the life cycle, i.e. egg, miracidium, sporocyst, cercaria, schistosomulum, and adult worm, by a modified stem-loop reverse transcribed polymerase chain reaction (RT-PCR) method developed in our laboratory. The expression patterns of these miRNAs were highly stage-specific. In particular, sja-miR-71 and sja-bantam expression reach their peaks in the cercaria stage and then drop quickly to the nadirs in the schistosomulum stage, following penetration of cercaria into a mammalian host.

**Conclusions:**

Authentic miRNAs were identified for the first time in *S. japonicum*, including a new schistosome family member. The different expression patterns of the novel miRNAs over the life stages of *S. japonicum* suggest that they may mediate important roles in Schistosome growth and development.

## Introduction

Noncoding RNAs (ncRNAs) of approximately 22 nucleotides (nt) in length are becoming increasingly recognized as important regulators of gene expression in animals, plants, and viruses [1]. One class of these short RNAs, microRNAs (miRNAs), inhibits gene expression through specific base-paring with target mRNAs. Mature miRNAs are produced through two sequential cleavages of longer precursors, which typically contain a stem-loop structure, by the miRNA processing enzymes, Drosha (which acts in the nucleus) and Dicer (which acts in the cytoplasm). These miRNAs combine with the Argonaut protein and other molecules to form a complex called RNA-induced silencing complex (RISC). RISC-bound miRNAs bind to their target mRNA through base pairing with the seed sequence, a section of the miRNA located at the 5′ end that includes 2–8 nucleotides, and either cleave the molecule or repress translation, thereby exerting post-transcriptional control of gene expression [2]. The seed sequence is an important component in miRNAs. It is thought that these nucleotides initiate a rapid zip up of the miRNA/mRNA duplex to overcome thermal hindrance, allowing further annealing of the miRNAs to the target sites and thermodynamic stabilization of the complex [3].

The first characterized endogenous miRNAs were lin-4 and let-7, both of which were shown to act in the pathway controlling the timing of larval development in *C. elegans* [4], [5]. Since then, numerous miRNAs have been identified in many organisms. Indeed the recent public miRNA registry (miRBase release11.0 at http://microRNA.sanger.ac.uk), which includes animal, plant, even virus miRNAs, contained 6396 entries at the time this manuscript was prepared. Indeed, the total number of miRNAs is estimated to represent approximately 1% of all genes in each genome, implying that a myriad of miRNAs and their roles in cellular processes such as development and size control of organisms, remain to be discovered [6], [7].

The platyhelminthe schistosome parasites (flatworms) cause schistosomiasis, which seriously affects 200 million people worldwide. The schistosome species that infect humans include *Schistosoma mansoni*, *Schistosoma haematobium*, and *Schistosoma japonicum*. In China, Schistosomiasis japonica remains a major public health problem. Humans can become infected by wading or bathing in contaminated water. Schitosomes have a complex life cycle, alternating between the definitive mammalian host and an intermediate freshwater molluscan host.

Schistosomes have haploid genomes estimated to be approximately 270 MB, arrayed on seven pairs of autosomes and one pair of sex chromosomes [8]. Although a schistosome genome has yet not been sequenced in its entirety, several hundred thousand schistosome expressed sequence tags (ESTs) and genome survey sequences are represented in GenBank, Sanger center, Shanghai LSBI, and some private databases. A unique repertoire of genes whose expression differs with life cycle stage and sex indicates that schistosomes have a complex gene regulation pattern [9]. Genomics and transcriptomics studies have revealed several ESTs in schistosomes with homology to Dicer and Argonaut, protein components of the miRNA silencing pathway [10]. Recently, the structure and expression of the Dicer gene of *S. mansoni* was characterized [11]. Furthermore, several studies have shown that schistosomes possess RNA interference (RNAi) molecular machinery, and that the addition of exogenous double-stranded RNAs can suppress target gene expression [12], [13]. Four putative miRNA candidates were predicted by bioinformatics methods [14].

In light of the evidence implicating small RNA species in gene regulation in flatworms, here we investigated potential miRNAs within *S. japonicum*. To this end, we constructed a library of size-fractionated RNAs from *S. japonicum* adult worms and analyzed 227 RNA sequences from a total of 106 insert-containing clones randomly selected from the library. From the abundant and diverse population of small RNAs observed in *S. japonicum*, potential miRNAs with flanking sequences that were predicted to fold into a stem-loop precursor structure were further detected by Northern blot. We further utilized a highly sensitive and specific stem-loop RT-PCR for quantitative analysis of the expression patterns of these novel miRNAs across the life span of *S. japonicum.*

## Materials and Methods

### Parasites

Adult worms and eggs of *S. japonicum* were isolated from the hepatic portal system and mesenteric veins of infected rabbits or mice at 6–7 weeks post infection. Male and female adult worms were manually separated under a light microscope. Hepatic schistosomula were isolated from the portal system and from mesenteric veins of infected mice at 2 weeks post-infection. All procedures performed on animals within this study were conducted in accordance with and by approval of the Internal Review Board of Tongji University School of Medicine. Cercariae of *S. japonicum* were shed from the lab-infected intermediate host, the snail *O. hupensis hupensis*, with hatched miracidia provided by National Institute of Parasitic Disease, Chinese Center for Disease Control and Prevention. After collection, all freshly isolated samples were washed three times with 1×PBS (pH 7.4) and were immediately used for extraction of total RNA or stored in liquid nitrogen until being subjected to further analysis.

### RNA isolation

Total RNA from samples from the 6 different life cycle stages of *S. japonicum* (egg, miracidium, sporocyst, cercaria, schistosomulum and adult worm) were extracted using Trizol® reagent (Invitrogen) according to the manufacturer's protocol with one modification. Namely, after addition of isopropanol, the RNA extract was incubated for at least 2 h at −20°C (instead of 5 min at room temperature) to enhance precipitation of low-molecular-weight (LMW) RNAs. Following a wash with 80% ethanol, RNA was re-suspended in DEPC-treated water or formamide (Sigma) and stored at −80°C.

### Small RNA isolation and library preparation

The cloning and analysis of small RNAs was performed essentially as described previously [15], [16]. RNA samples were enriched for LMW species by removal of high molecular weight mRNA and rRNA. Total RNA (600 µg) from a mixture of male and female adult worms was combined with 50% PEG 8000 to a final concentration of 5%, and 5 M NaCl to a final concentration of 0.5 M, and the LMW RNAs were precipitated by adding 2.5 volumes of 100% EtOH at −20°C for at least 2 h. The LMW RNAs were then resolved by electrophoresis on denaturing 15% polyacrylamide gel with 8 M urea, and short RNAs (18 to 26 nucleotides in length) were excised and eluted in 0.3 M NaCl at 4°C for approximately 16 h. The eluted short RNAs were precipitated in ethanol, resuspended in RNase-free water and ligated sequentially to a 3′ (5′-pacuGTAGGCACCATCAAx-3′) RNA/DNA adaptor (lower-case nucleotides, ribonucleotides; p, phosphate group; x, C6-NH2 modification) and then to a 5′ (5′-ATCGTaggcacctgaaa-3′) adaptor. For the ligation, the short RNAs were first dephosphorylated using Shrimp Alkaline Phosphatase (Takara), and then ligated to the 3′ RNA/DNA adaptor using T4 RNA Ligase (Takara) according to the manufacturer's instructions. Further ligation to the 3′ end of the 3′ adaptor was blocked by the C6-NH2 group on the 3′ end of the adaptor oligonucleotide. After PAGE purification of the ligated products and rephrosphorylation of the 5′ terminus, ligation of the 5′ DNA adaptor was performed. The final ligation product was ethanol precipitated, resolved on denaturing 15% polyacrylamide, eluted from the gel, ethanol precipitated, and used as a template in a reverse transcription reaction using an RT primer (5′-ATTGATGGTGCCTAC-3′) which is complementary to part of the 3′ adaptor sequence (underlined). Resulting cDNAs were amplified by PCR using antisense (5′-ATTGATGGTGCCTACAG-3′) and sense (5′-ATCGTAGGCACCTGAAA-3′) primers containing internal BanI restriction sites (underlined), based on the sequences of the 5′ adaptor and the RT primer. Resulting PCR products were purified, digested with BanI, concatenated, then ligated into pGEM-T Easy vector (Promega). Following transformation, plasmids were purified from ampicillin-resistant colonies and sequenced.

### Computational analyses

After masking of vector and adaptor sequences and removal of redundancy, the inserts of sizes between 18 and 26 nt obtained from the *S. japonicum* small RNAs library, were used in BLAST searches to identify candidate *S. japonicum* miRNAs. We screened the databases of *S. japonicum* transcriptome (http://www.ncbi.nih.gov/Genbank/index.html), and entire collection of unassembled genomic sequence reads (http://lifecenter.sgst.cn/sj.do). The *S. mansoni* genome database (http://www.sanger.ac.uk/Projects/S_mansoni) was also used for the supplementary annotation analysis of cloned sequences by using WU-BLAST with default parameters and a non-stringent cutoff of E<1.8[17]. Sequences containing more than 3 mismatches with any reported sequence were discarded from further analysis. After removal of the contaminating rRNA and tRNA sequences and sequences that perfectly matched spliced ESTs, the remaining small RNAs were further analyzed for predicted secondary structure. The genomic regions containing sequences homologous to the *S. japonicum* miRNA candidate sequences with approximately 100 bp of flanking sequence were extracted and analyzed using RNA-fold software such as Mfold (http://www.bioinfo.rpi.edu/applications/mfold) and Vienna RNAfold (http://rna.tbi.univie.ac.at/cgi-bin/RNAfold.cgi) for the potential to adopt a stem-loop conformation typical of pre-miRNAs with a folding free energy of at least 25 kcal/mole (Δ*G*°_folding_≤−25 kcal/mol). The candidate sequences were then screened against a database of known miRNAs (http://www.sanger.ac.uk/Software/Rfam/mirna) to compare our candidate *S. japonicum* miRNAS to miRNAs in other species. In order to further verify candidate miRNAs, mature miRNAs and pre-miRNAs from *S. japonicum* and *S. mansoni* genomes were subjected to family alignment processing using the ClustalW2 program (http://www.ebi.ac.uk/Tools/clustalw2/index.html).

### Northern blot analysis

As a verification step and to provide size and sequence confirmation of candidate miRNAs, total RNA or LMW RNAs extracted from a mixture of male and female *S. japonicum* adult worms were separated by electrophoresis on a 15% polyacrylamide gel under denaturing conditions, and transferred to Hybond-N nylon membrane (Pall). After a brief rinse in 2×SSC, the filter was blotted dry, and the RNA was uv-crosslinked to the filter. The filter was pre-hybridized for 2 h at 37°C in hybridization solution (Beijing Biodev-tech. scientific&technical co. ltd, China), then incubated with approximately 100 ng/ml biotin-labeled oligonucleotide probe in hybridization solution for 8–12 h at 37°C. We probe sequences that were complementary to the candidate miRNAs identified from the *S. japonicum* small RNA library. Each oligo were labeled one biotin moieties in 5′-end (Invitrogen, Shanghai). After hybridization, filters were washed twice in 2×SSC and 0.1% SDS at room temperature for 10 min and twice in 0.1×SSC and 0.1% SDS at 45°C for 20 m. Filters were incubated in blocking buffer (Pierce) for 15 min, then streptavidin-HRP conjugate (Pierce) was added to the blocking buffer (Pierce) at a 1∶300 dilution and incubated for 15 min at room temperature. Filters were washed 4 times with washing buffer (Pierce) for 5 min, then incubated in equilibration buffer (Pierce) for 5 min. For detection, the filters were incubated in enhanced luminal-based chemiluminescent substrate (Pierce) for 15 min. Finally, the membranes were covered with plastic wrap and exposed to X-ray film for 20 min at room temperature for signal detection.

### Analysis of miRNA expression

Because of their small size, detection of these miRNAs by traditional methods is technically demanding. The stem-loop reverse transcription primers provide more specificity and sensitivity than linear primers owing to the base stacking and spatial constraints of the stem-loop structure. Therefore, stem-loop real-time reverse transcriptase polymerase chain reaction (RT-PCR) was used to quantitate miRNA expression across the life cycle of *S. japonicum*. The primers for these reactions were designed according to Chen et al. [18] and Tang et al. [19] and required the design of an RT primer with a stem-loop structure, and a set of primers for the subsequent quantitative PCR amplification. In order to increase the specificity of this method, the sequence length of the stem-loop RT primer to individual miRNAs was extended to 8 nucleotides complementary to the 3′ end of the miRNA. Moreover, an additional 12 nucleotides were added to each forward primer to increase the melting temperature. In addition, specific fluorescent probes were replaced by cost-effective SYBR Green in the real-time PCR experiments. The modified method was performed as follows: The stem-loop RT primer was used to reverse transcribe the miRNA molecule using total RNA isolated from *S. japonicum* adult worm. The RT product was then amplified using a miRNA-specific forward primer and a universal reverse primer. The primers used in the RT and subsequent quantitative PCRs are specified in Table S1. The common reverse primer is based on a sequence from the loop portion of an RT stem-loop primer.

The RT reactions contained 2 µg total RNA isolated from the tissue samples from each of the six different life cycle stages of *S. japonicum*, 50 nM each individual stem-loop RT primer, 2 U RNase inhibitor, 5 U M-MLV reverse transcriptase (Takara), and 0.5 µM dNTP. The reaction parameters were as follows: 16°C for 30 min, 42°C for 30 min, 70°C for 15 min, and holding at 4°C. To generate the cDNA template for the endogenous control quantitative PCR reactions, first strand cDNA was synthesized using oligo(dT) in the presence of 2 µg of total RNA isolated from the tissue samples from each of the six life cycle stages. The synthesis reaction conditions were as follows: 42°C for 30 min, 70°C for 15 min, then hold at 4°C.

Real-time quantitative PCR was performed using an Applied Biosystems 7300 detection system in a 15 µl reaction. All reactions were carried out in duplicate. For quantitation of the three miRNAs, the PCRs were carried out in a total volume of 15 µl, including 1 µl of RT product for each miRNA, 1×SYBR Green I Mastermix, 0.5 µM specific forward primer, and 0.5 µM common reverse primer. For the endogenous control, α-tubulin, 1 µl of cDNA synthesized with oligo(dT) was used as the template. The α-tubulin primers were as follows: forward 5′-GTACATGTTGGTCAAGCTGGTGT-3′ and reverse 5′-AGTTCGCACTTCATCCACTACAGT-3′. The quantitative RT-PCR reaction conditions were as follows: 95°C for 10 min, followed by 40 cycles of 95°C for 15 s, 60°C for 1 min. The threshold cycle (Ct) was defined as the cycle number at which the fluorescence intensity passes a pre-determined threshold. In our analyses, quantification of each miRNA relative to α-tubulin was calculated using the following equation [20]: N = 2^−ΔCt^, ΔCt = Ct_miRNA_−Ct_α-tubulin_. Dissociation curves were generated and 8% PAGE electrophoresis was performed for each real-time RT-PCR to verify the amplification of only the desired product.

## Results

### Cloning of short RNAs from *S. japonicum*

In order to identify the miRNAs in *S. japonicum*, we generated two libraries of small (18–26 nt) RNA species, one from cercaria and the other from adult worm. When the cercaria library sequences were further analyzed using the annotated gene database at GenBank and http://lifecenter.sgst.cn/sj.do, all sequences proved to be fragments of rRNA or tRNA sequences (data not shown). Therefore, we proceeded to analyze only the library cloned from *S. japonicum* adult worm.

A total of 106 insert-containing clones randomly selected from the library yielded 227 unique sequences, which indicated that each concatamer contained ∼2 small RNA sequences. The sequences ranged in length from 18 to 26 nt, which is consistent with the size of known miRNAs processed by Dicer. As shown in Table S2, BLAST analysis against the GenBank, Sanger center, and Shanghai LSBI databases containing the entire collection of unassembled genomic sequence reads and transcriptomes of *S. japonicum* and *S. mansoni* indicated that among the isolated small RNAs, 65.63% were rRNA, 3.52% were mRNA, and 24.24% were ncRNAs. The 55 ncRNAs were further analysed for the potential miRNAs.

### Analysis of potential *S. japonicum* miRNAs

**Figure 1:**
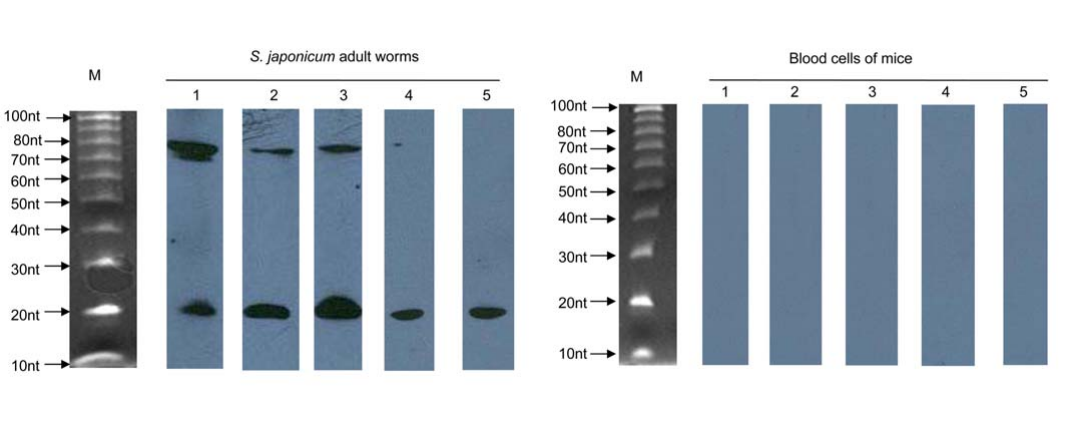
Detection of miRNAs by Northern blot. RNAs (10 µg) isolated from *S. japonicum* adult worms or blood cells of mice (as negative control) were separated on a 15% denaturing PAGE gel and transferred to a nylon membrane. Membranes were incubated with five different biotin-labeled probes (1: sja-let-7, 2: sja-miR-71, 3: sja-bantam, 4: sja-miR-125 and 5: sja-miR-new1). M: 10 bp DNA marker. the ladder was denatured in the same way as the RNAs done so that the ladder run as single stranded DNA.

One of the important features that distinguish miRNAs from other endogenous small RNAs is the ability of the miRNA precursor sequences to adopt a hairpin structure [2]. To determine whether these cloned small RNA sequences from *S. japonicum* are actual miRNAs, we first removed contaminating rRNA, tRNA, and EST sequences. A nonredundant set of 12 sequences out of the 55 ncRNA clones were predicted to be capable of forming stem-loop structures characteristic of miRNA precursors. These 12 candidate sequences were further examined by Northern blot to confirm their size and sequence. Northern blots of total RNA from a mixture of male and female *S. japonicum* adult worms were hybridized with biotin-labeled probes of the twelve candidate sequences. Five of these twelve probes gave a hybridization signal that was characteristic of miRNA (∼22 nt mature miRNA and /or ∼70 nt pre-miRNA) (Figure 1). Unless due to the lesser sensitivity of chemiluminescent detection relative to the radioactive method, the absence of signal in lanes 4 and 5 of the Northern blot (Figure 1) appears to suggest that the two precursors were expressed at low levels.

**Figure 2:**
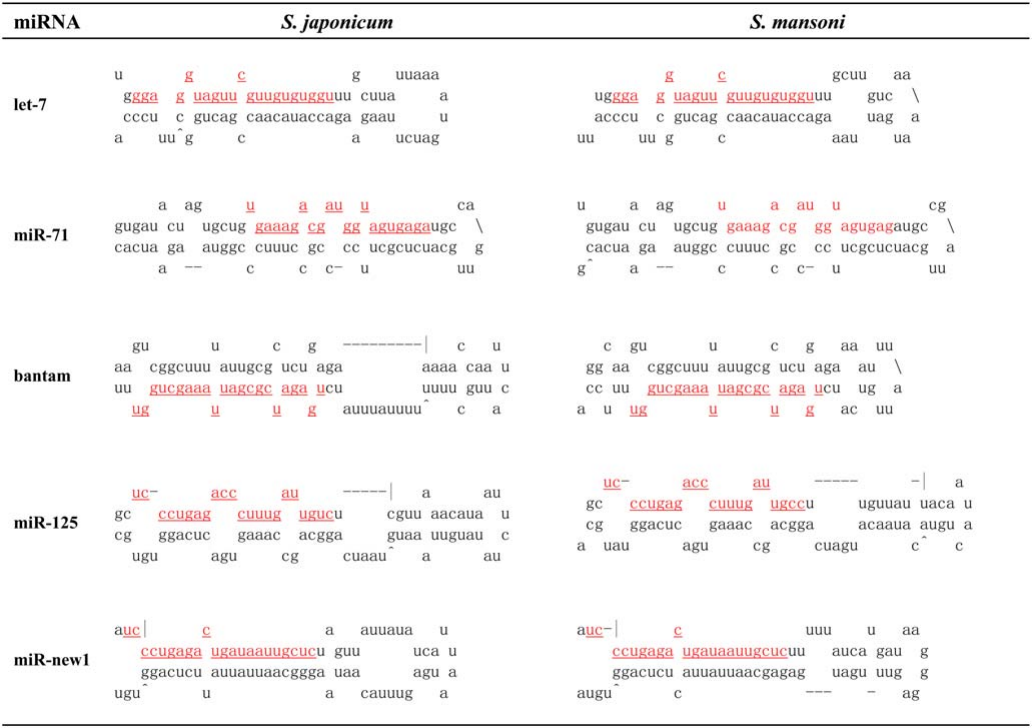
Predicted stem-loop structures for the miRNA precursors of *S. japonicum* and *S. mansoni*. The mature miRNA sequence is shown in red and underlined. The actual size of each putative precursor was not identified experimentally and may be slightly shorter or longer than represented. Mir-bantam and mir-125 precursor from *S. japonicum* showed large bulges in their secondary structure predicted by Mfold RNA-fold software. However, when using Vienna RNAfold method for structure prediction, mir-125 precursor showed acceptable bulges in their secondary structure while the bugles of bantam precursor remained (see figure S1).

Seven of the twelve potential sequences were excluded for the following reasons: four candidates gave multiple bands on the Northern blot that were greater than 100 nt, which is beyond the size limitation for miRNAs; and three candidates showed no signal by Northern blotting and thus will require additional validation. It is possible that these sequences are authentic miRNAs that are expressed at low levels. The remaining five candidate sequences were demonstrated to be authentic miRNAs. Their sequences and predicted secondary structures are shown in Table 1 and Figure 2. Very close homologs in other species can be annotated as miRNA homologs without experimental validation if they have phylogenic conservation and predicted fold-back precursor secondary structure [21]. Pre-miRNA alignments with flanking sequences between *S. mansoni* and *S. japonicum* showed high conservation (Figure S2), indicating that these miRNAs should also exist in *S. mansoni*.

**Table 1:** Sequence of the three novel miRNAs identified in adult *S. japonicum* and their locations within the published chromosomal sequence.

miRNA	Sequence	Size (nt)	*S. japonicum* contig (LSBI, Shanghai)^a^	*S.mansoni* shortgun reads (Sanger)^b^	Clones^c^	Δ*G*°_folding_ (kcal/mol^e^)
sja-let-7	GGAGGUAGUUCGUUGUGUGGU	21	CNUS0000067197: 5856–5876	shisto12670f07: 651–671	5	−30.8
sja-miR-71	UGAAAGACGAUGGUAGUGAGA	21	CNUS0000007682(-)^d^: 3100–3120	shisto8708d10: 353–372	1	−34.5
sja-bantam	UGAGAUCGCGAUUAAAGCUGGU	22	CNUS0000021739: 2223–2244	shisto5226g02(-)^d^: 325–346	6	−22.9
sja-miR-125	UCCCUGAGACCCUUUGAUUGUC	22	CNUS0000024724:7691–7712	Smp_contig001766:3162–3183	2	−25.6
sja-miR-new1	UCCCUGAGACUGAUAAUUGCUC	22	CCON0000000380 (-)^d^:353325–353346:15–36	shisto8125f02.p1k	4	−29.2

a location of the miRNA sequence within the published chromosomal sequence of *S. japonicum*.

b location of the miRNA sequence within the published chromosomal sequence of *S.mansoni*.

c the number of clones pf each type found in our library of *S. japonicum* small RNAs.

d the “-” notation indicates miRNA sequence produced from reported chromosomal sequence.

e the delta G indicated the stability of the pre-miRNA hairpin.

**Figure 3:**
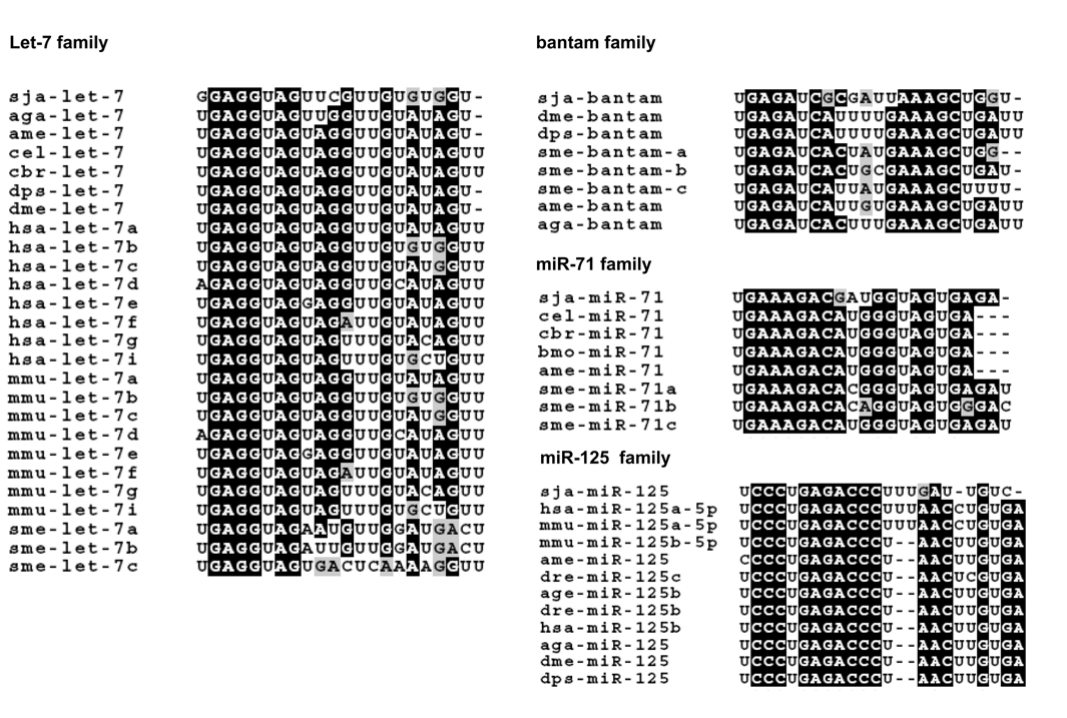
Alignments of miRNA sequences from *S. japonicum* and other organisms. Alignments of *S. japonicum* (sja) miRNAs with sequences from *S. mediterranea* (sme), *A. gambiae* (aga), *A. mellifera* (ame), *D. melanogaster* (dme), *D. pseudoobscura* (dps), *C. briggsae* (cbr), *C. elegans* (cel), *B. mori* (bmo), mouse (mmu), and human (hsa) were performed with ClustalW (http://www.ebi.ac.uk/Tools/clustalw2/index.html). The seed regions (typically the 5′ 2–8 nucleotides of the miRNA), which serve to anchor the miRNA to their mRNA targets, are identical across species.

Alignments with known miRNA sequences indicated that four of the five novel *S. japonicum* miRNAs belong to four different metazoan miRNA families, i.e. let-7, miR-71, bantam, and miR-125 (Figure 3, S3 and S4). As shown in Figure 3, the seed regions (typically the 5′ 2–8 nucleotides of the miRNA) which serve to anchor the miRNA to their mRNA targets are identical across species. The fifth novel *S. japonicum* miRNAs could not be matched any known miRNA sequences in miRBase and hence it was determined to belong to a new, previously not described family of miRNAs in schistosome. Hence, herein we have tentatively designated the five novel miRNAs from *S. japonicum* as sja-let-7, sja-miR-71, sja-bantam, sja-miR-125 and sja-miR-new1, respectively. Given the sequence-based evidence in support of these sequences as true miRNAs, we proceeded to compare the expression patterns of the *S. japonicum* miRNAs at various life stages of the parasite.

### Quantification of *S. japonicum* miRNA expression using a modified stem-loop RT-PCR method

We modified the stem-loop Taqman miRNA RT-PCR assay developed previously [18] to generate a modified stem-loop RT-PCR method for analysis of *S. japonicum* miRNA expression. Dissociation curves in the SYBR Green real-time RT-PCRs presented as a single, sharp narrow peak (Figure S5C). Analysis of a single band of appropriate size (approximately 65 bp) on 8% PAGE showed its specificity and sensitivity for detection of *S. japonicum* miRNAs (Figure S5A). No RT product was obtained from the “minus-RT” control in which reverse transcriptase was omitted from the reactions (data not shown). Furthermore, the RT reaction using host RNA isolated from total blood cells of rabbit and mice as the template also did not produce amplification (lane 1 and 2 in Figure S5B), further confirming that the three novel miRNAs are specific for *S. japonicum*.

To investigate the ability of the modified stem-loop RT-PCR method to discriminate between mature miRNAs and their precusors, various RNA templates were isolated from *S. japonicum* including 5 µg total RNA, ≤200 nt RNA (LMW RNA), miRNA precursors (60–100 nt) and mature miRNAs (18–26 nt). Each of isolated templates was purified from 5 µg total RNA by denaturing in a 15% polyacrylamide gel. Similar yields of amplification product were obtained from the reactions using total RNA and LMW RNA after 22 and 30 cycles. The amplification product was not detectable in the reactions after 22 cycles, but a small amount of product was observed after 30 cycles using isolated miRNAs precursors as a template (Figure S5D). These data indicate that the modified stem-loop RT-PCR method is highly specific for mature miRNAs.

In addition, we synthesized the sja-miR-71 and used this synthetic sja-miR-71 to measure dynamic range and sensitivity of the miRNA quantification. As shown in Figure S6, the log of target input and CT value showed excellent linearity when the dynamic range within 3 logs, suggesting that this assay can detect 1fM miRNA in the PCR reaction. At the same time, the CT value was also correlated to total RNA input (R2 = 0.99) over three orders of magnitude (ranged from 0.016 to 16 ng) (Figure S7). Although the sensitivity is lower compared to the previous procedure described by Chen, et al. [18], this modified stem-loop RT-PCR method should be enough sensitive to measure the expression of these miRNAs.

### Relation of *S. japonicum* miRNA expression to life cycle stage

Using the modified stem-loop RT-PCR method described above, we further endeavored to determine the timing of expression patterns of miRNA sja-let-7, sja-miR-71 and sja-bantam across the life span of *S. japonicum*. Table 2 shows expression levels of the three novel miRNAs during the six life stages of *S. japonicum*: egg, miracidium, sporocyst, cercaria, schistosomulum and adult worm. The detection was normalized to expression of the endogenous control, α-tubulin (Figure S8). Sja-let-7 expression was lowest in the miracidium stage, increased during the sporocyst stage, peaked during the cercaria stage, and then proceeded to decrease for the remainder of the organism's lifespan within the mammalian host. Interestingly, both sja-miR-71 and sja-bantam showed maximum expression in cercaria (infective stage of the parasite) and their expression then dropped quickly to nadir levels in schistosomulum following penetration of the cercaria into its host. Expression of sja-miR-71 and sja-bantam were 1000-fold and 500-fold higher in cercaria than that in schistosomulum, respectively. The expression levels of these three miRNAs did not differ between female and male adult worms.

**Table 2:** Expression levels of the three novel miRNAs across the lifespan of *S. japonicum*.

miRNA	egg	miracidium	sporocyst	cercaria	schistosomulum	male adult worm	female adult worm
sja-let-7	1.15±0.96	0.02±0.02	0.24±0.22	5.92±4.02	0.60±0.41	0.75±0.35	0.59±0.13
sja-mir-71	8.68±5.06	3.21±2.17	4.57±0.79	1257.92±565.47	0.91±0.34	1.93±1.41	1.65±0.36
sja-bantam	4.50±2.67	1.69±0.91	1.59±1.08	295.44±361.15	0.59±0.11	1.01±0.78	2.24±0.46

The expression levels of three novel miRNAs were normalized to the expression levels of endogenous α-tubulin. The data represent the means and standard errors for triplication reactions independently.

## Discussion

MiRNA is recognized as important regulators of gene expression through specific base-paring with the target mRNA. Although a large number of miRNA have been identified from plants to mammals, it is not known whether human parasites including schistosome encode miRNAs in addition to mosquito. The schistosome parasite is a eukaryotic organism. There has been a strong suspicion that miRNAs may exist in the flatworm based on the following observations: (1) genomics and transcriptomics studies have revealed that several ESTs in schistosomes have homology to Dicer and Argonaut that are protein components of the miRNA silencing pathway [10]; (2) the structure and expression of the Dicer gene of *S. mansoni* was recently characterized [11]. (3) schistosomes possess RNAi molecular machinery and the addition of exogenous double stranded RNAs can suppress target gene expression [12], [13]. (4) Heart-stirringly, four putative miRNA candidates were predicted by Hertel *et al* using bioinformatics methods [14].

In this study, we firstly identified five authentic miRNAs in *S. japonicum* by constructing and screening parasite cDNA library of size-fractionated RNAs: sja-let-7, sja-miR-71, sja-bantam, sja-miR-125 and sja-miR-new1. Analysis of sja-let-7, sja-miR-71 and sja-bantam expression by stem-loop RT-real time PCR revealed highly stage-specific expression patterns. Importantly, the expression of sja-miR-71 and sja-bantam peaked in cercaria and then decreased quickly to their lowest levels in schistosomulum, following host infection. These findings suggest that these miRNAs are involved in schistosome infection, growth and development. The identification and examination of the expression and functions of these miRNAs opens up a new field of study in this important parasite pathogen.

The quantification of miRNAs at various life cycle stages is essential for elucidating their functions in gene regulation. Conventional methods such as Northern blot may not have sufficient sensitivity to detect low abundance miRNAs. A stem-loop Taqman miRNA RT-PCR assay was recently developed for miRNA detection based upon a RT reaction initiated by a stem-loop primer [18]. However, this method has limitations. Firstly, it was developed primarily for analysis of miRNAs in mammals. Secondly, the protocol calls for costly specific fluorescent probes. For our experiments, we required a high level specificity of detection to discriminate among miRNAs derived from the parasite, mammalian and intermediate freshwater molluscan host. Therefore, we developed a modified stem-loop RT-PCR method for analysis of *S. japonicum* miRNA expression. Our experiments demonstrated that this more cost-efficient method enables high specificity, sensitive detection of miRNAs in S. *japonicum*. Importantly, it provides precise discrimination between *S. japonicum* and host miRNAs. A similar method recently described by Varkonyi-Gasic et al. [22] also showed high specificity and sensitivity.

The over-representation of degraded rRNAs in the pool of short RNAs in the *S. japonicum* library severely hinder discovery of miRNAs. As shown in Table S2, the fragments of 28S rRNA comprised approximately one half of the total short RNAs isolated from *S. japonicum*. Although we repeated the cloning process several times, the very high percentage of molecules of rRNA origination remained (data not shown). The low percentage of molecules of mRNA origination rules out the possibility of external degradation during the RNA isolation and cloning procedures. The phenomenon known as the large rRNA subunit “nick *in vivo*” has previously been reported in schistosome [23]. Analysis by 1% agarose gel electrophoresis indicated that RNA extracted from *S. japonicum* adult worm showed only one major band slightly larger than 18S, which was distinct from the two major bands (28S and 18S) typically seen in other species. This degradation of the 28S large rRNA subunit may be the source of the large proportion of rRNA fragments cloned into the small RNA library.

miRNAs regulate a variety of developmental and physiological processes. Most miRNAs are expressed in a developmental or organ-specific manner, or both, which provide a few hints about their functions [4], [5], [24], [25]. As a new class of sequence-specific regulators of gene expression, miRNAs may take part in a regulatory network along with transcription factors and growth factors to control the development and differentiation of the platyhelminthe *S. japonicum*.

The role of let-7 during metamorphosis in Drosophila and in C. elegans development is well documented for *C. elegans* and *D. melanogaster* [4]. The let-7 miRNA is active during the last larval stage in *C. elegans*, promoting the transformation from larva to adult, and is also highly expressed in the late third instar larval stage of *D. melanogaster* when a pulse of the ecdysone triggers puparium formation and onset of metamorphosis [26]. The expression pattern during the life cycle of *S. japonicum* indicates that sja-let-7 might take part in the transformation from miracidium to sporocyst in the snail intermediate host. Bantam miRNAs are known regulators of both proliferation and apoptosis, and target the proapoptotic gene *hid* [27]. Reduced growth rate observed in the third instar wing disc of drosophila expressing mutant bantam genes could be due to reduced rates of cell proliferation or increased apoptosis, or both. Removing one copy of the endogenous bantam gene in drosophila has been shown to enhance, and conversely overexpression of bantam has been shown to suppress, the level of *hid*-induced apoptosis in the eye [27]. Recent studies have revealed that bantam overexpression mitigates neurodegeneration induced by the pathogenic polyglutamine protein Ataxin-3, which is involved in the human disease spinocerebellar ataxia type 3 (SCA3) [28]. Based on these studies, we would expect sja-bantam to take part in developmental processes throughout the lifespan of *S. japonicum* by regulating cell proliferation and apoptosis. Thus far, no studies concerning the function of miR-71 family miRNAs have been reported. It has been difficult to construct a detailed 3′-UTR library from the schistosoma transcriptome because of an incomplete EST sequence, which has greatly inhibited extensive computational prediction of these miRNAs targets.

Further work aimed at identifying the target mRNAs of these novel miRNAs will be needed to elucidate the functions of newly identified miRNAs in *S. japonicum*. Untangling *S. japonicum* physiology can have important ramifications for efforts to control schistosmiasis, which remains a substantial challenge in endemic areas due to the difficulty of eradicating the snail intermediate hosts. Clarifying the role of miRNAs in the parasite's growth, development and ability to infect mammalian hosts will be particularly important given its complex life cycle, involving several distinct developmental stages of vertebrate and invertebrate hosts and a unique repertoire of genes expressed at different life cycle stages. Elucidation of schistome gene regulation will enable dissection of the biological basis of antigenic diversity, infectivity, and pathology, and will provide the best prospects for identifying new drug targets and vaccine candidates.

## Supporting Information

Table S1 Sequences of the primers used for stem-loop RT-PCR.Stem-loop RT primers were designed according to Chen et al. [17], but the priming regions were extended to 8 nucleotides complementary to the 3′ end of individual miRNA. Sequences were shown in italic, bold and underlined. To increase the melting temperature, an additional 12 nucleotides (bold and underlined sequences) were added to each forward primer. The common reverse primer which comes from the loop portion of an RT stem-loop primer was shown in bold and italic. All these common sequences were screened from the databases of S. japonicum transcriptome and genome, and S. mansoni genome as well to eliminate the homology.
(0.03 MB DOC)

Table S2 Composition in percentage of the 227 total sequences analyzed from the library of S. japonicum small RNAs. The annotation was based on information from the databases of S. japonicum transcriptome (http://www.ncbi.nih.gov/Genbank/index.html), the entire collection of unassembled genomic sequence reads (http://lifecenter.sgst.cn/sj.do) and the S. mansoni genome (http://www.sanger.ac.uk/Projects/S_mansoni). a, the sequence of 18 clones contained 5 miRNAs that matched the criteria of miRNA and were verified by Northern blot. b, sequences of the clones can form stem-loop structures of miRNA precursors but were not verified by Northern blotting. c. Sequences of the clones contain more than three mismatches with the reported genome and transcriptome sequence in GenBank, Sanger center, Shanghai LSBI libraries.
(0.03 MB DOC)

Figure S1 Predicted stem-loop structures for the mir-bantam and mir-125 precursors of S. japonicum and S. mansoni using Vienna RNAfold method. Mir-125 precursor from both S. japonicum and S.mansoni genome showed acceptable bulges in their secondary structure by Vienna RNAfold method. The bugle in the secondary structure of bantam precursor from S.mansoni was disappeared while the bugle from S. japonicum genome remains.
(0.92 MB TIF)

Figure S2 Alignments of S. japonicum miRNAs precursors with S.mansoni. The sequences of mature microRNAs are boxed.
(1.19 MB TIF)

Figure S3 Sequence alignments of pre-miRNAs in each miRNA family. Abbreviation is the same as Figure 3.
(4.50 MB TIF)

Figure S4 Phylogeny analysis of four miRNAs precursors. Abbreviation is the same as Figure 3.
(0.74 MB TIF)

Figure S5 The specificity of stem-loop RT-PCR for S. japonicum miRNA quantitation. (A) Electrophoresis of the miRNA RT-PCR products on 8% PAGE showing bands of the anticipated size (65 bp). (B) Electrophoresis of the miRNA RT-PCR products on 8% PAGE showing no amplification using host RNA template. Lane 1: total RNA isolated from total blood cells of rabbit, lane 2: total RNA isolated from total blood cells of mice, lane 3: total RNA isolated from S. Japonicum, lane S: stem-loop RT-PCR reactions using the RNA of S. Japonicum adult worm with the mice mmu-let-7c specific primers. (C) Dissociation curves for the three duplicate RT-PCR reactions showing specificity of the reactions. (D) The specificity of the stem-loop RT-PCR assay in detecting mature miRNA expression. Four different RNA templates (lanes 1–4) were subjected to stem-loop RT PCR using the indicated stem-loop RT primer, then electrophoresed on 8% PAGE. Lane 1, 5 µg total RNA, lane 2, low molecular weight (LMW) RNA purified from 5 µg total RNA , lane 3, miRNA precursors (60–100 nt) isolated from 5 µg total RNA on denaturing 15% polyacrylamide gel, lane 4, mature miRNA (18–26 nt) isolated from 5 µg total RNA on denaturing 15% polyacrylamide gel. PCR cycle numbers are indicated to the left.
(3.95 MB TIF)

Figure S6 Dynamic range and sensitivity of sja-miR-71 RT-QPCR assay using synthetic sja-miR-71 miRNA. (A) Amplification plot of synthetic sja-miR-71 miRNA over three orders of magnitude. Synthetic RNA input ranged from 1pM to 1000pM in PCR. (B) Standard curve of the sja-miR-71 miRNA.
(0.94 MB TIF)

Figure S7 Dynamic range of sja-miR-71 RT-QPCR assay using adult S. japonicum total RNA. (A) Amplification plot of sja-miR-71 miRNA over three orders of magnitude. Total RNA input ranged from 0.016 to 16 ng per RT reaction. (B) Correlation of total RNA input to the threshold of cycle (CT) values of sja-miR-71 miRNA assays.
(0.96 MB TIF)

Figure S8 The amplification plot of sja-let-7, sja-mir-71, sja-bantam and alpha tubulin. The cDNAs for QPCR were obtained from the RT reactions that used the same cercaria sample. The RT reactions and consequent real-time quantitative PCR were performed at the same conditions. 1, 2, 3 and 4 were the amplification plot of sja-mir-71, sja-bantam, sja-let-7 and alpha tubulin, respectively, the RT product of cercaria RNAs. 5, 6, 7 and 8 were the amplification plot of sja-let-7, sja-mir-71, alpha tubulin and sja-bantam, respectively, using the no-RT control.
(2.30 MB TIF)
