# Supplementary material for: Identification and Characterization of Novel MicroRNAs from Schistosoma japonicum
Source: PLoS One. 2008 Dec 24;3(12):e4034. doi: 10.1371/journal.pone.0004034 (PMC2603315; doi:10.1371/journal.pone.0004034)
Supplement: Table S1 — Sequences of the primers used for stem-loop RT-PCR.Stem-loop RT primers were designed according to Chen et al. [17], but the priming regions were extended to 8 nucleotides complementary to the 3′ end of individual miRNA. Sequences were shown in italic, bold and underlined. To increase the melting temperature, an additional 12 nucleotides (bold and underlined sequences) were added to each forward primer. The common reverse primer which comes from the loop portion of an RT stem-loop primer was shown in bold and italic. All these common sequences were screened from the databases of S. japonicum transcriptome and genome, and S. mansoni genome as well to eliminate the homology. (0.03 MB DOC) [file pone.0004034.s001.doc]

| sja-let-7 | Sequence | GGAGGUAGUUCGUUGUGUGGU |
| --- | --- | --- |
|  | RT stem-loop primer | GTCGTATCCAGT***GCAGGGTCCGAGGTATTC***GCACTGGATACGAC***ACCACACA*** |
|  | Forward primer | **ATCGTACGTGGG**GGAGGTAGTTCGTTG |
| sja-miR-71 | Sequence | UGAAAGACGAUGGUAGUGAGA |
|  | RT stem-loop primer | GTCGTATCCAGT***GCAGGGTCCGAGGTATTC***GCACTGGATACGAC***CTCACTAC*** |
|  | Forward primer | **ATCGTACGTGGG**TGAAAGACGATGGT |
| sja-bantam | Sequence | UGAGAUCGCGAUUAAAGCUGGU |
|  | RT stem-loop primer | GTCGTATCCAGT***GCAGGGTCCGAGGTATTC***GCACTGGATACGAC***ACCAGCTT*** |
|  | Forward primer | **ATCGTACGTGGG**TGAGATCGCGATTAAA |
| Common reverse primer | | GCAGGGTCCGAGGTATTC |
