# Supplementary material for: Identification and Characterization of Novel MicroRNAs from Schistosoma japonicum
Source: PLoS One. 2008 Dec 24;3(12):e4034. doi: 10.1371/journal.pone.0004034 (PMC2603315; doi:10.1371/journal.pone.0004034)
Supplement: Table S2 — Composition in percentage of the 227 total sequences analyzed from the library of S. japonicum small RNAs. The annotation was based on information from the databases of S. japonicum transcriptome (http://www.ncbi.nih.gov/Genbank/index.html), the entire collection of unassembled genomic sequence reads (http://lifecenter.sgst.cn/sj.do) and the S. mansoni genome (http://www.sanger.ac.uk/Projects/S_mansoni). a, the sequence of 18 clones contained 5 miRNAs that matched the criteria of miRNA and were verified by Northern blot. b, sequences of the clones can form stem-loop structures of miRNA precursors but were not verified by Northern blotting. c. Sequences of the clones contain more than three mismatches with the reported genome and transcriptome sequence in GenBank, Sanger center, Shanghai LSBI libraries. (0.03 MB DOC) [file pone.0004034.s002.doc]

| Class of RNA | | **Number of clones** | **Percentage** |
| --- | --- | --- | --- |
| rRNA | |  |  |
|  | 28S rRNA | 102 | 44.93% |
|  | 18S rRNA | 47 | 20.70% |
| mRNAs | | 8 | 3.52% |
| ncRNAs | |  |  |
|  | identified miRNA^a^ | 18 | 7.93% |
|  | potential miRNA^b^ | 8 | 3.52% |
|  | Unidentified | 29 | 12.78% |
| not matched^c^ | | 15 | 6.61% |
