# Supplementary figures and images for: Identification and Characterization of Novel MicroRNAs from Schistosoma japonicum
Source: PLoS One. 2008 Dec 24;3(12):e4034. doi: 10.1371/journal.pone.0004034 (PMC2603315; doi:10.1371/journal.pone.0004034)

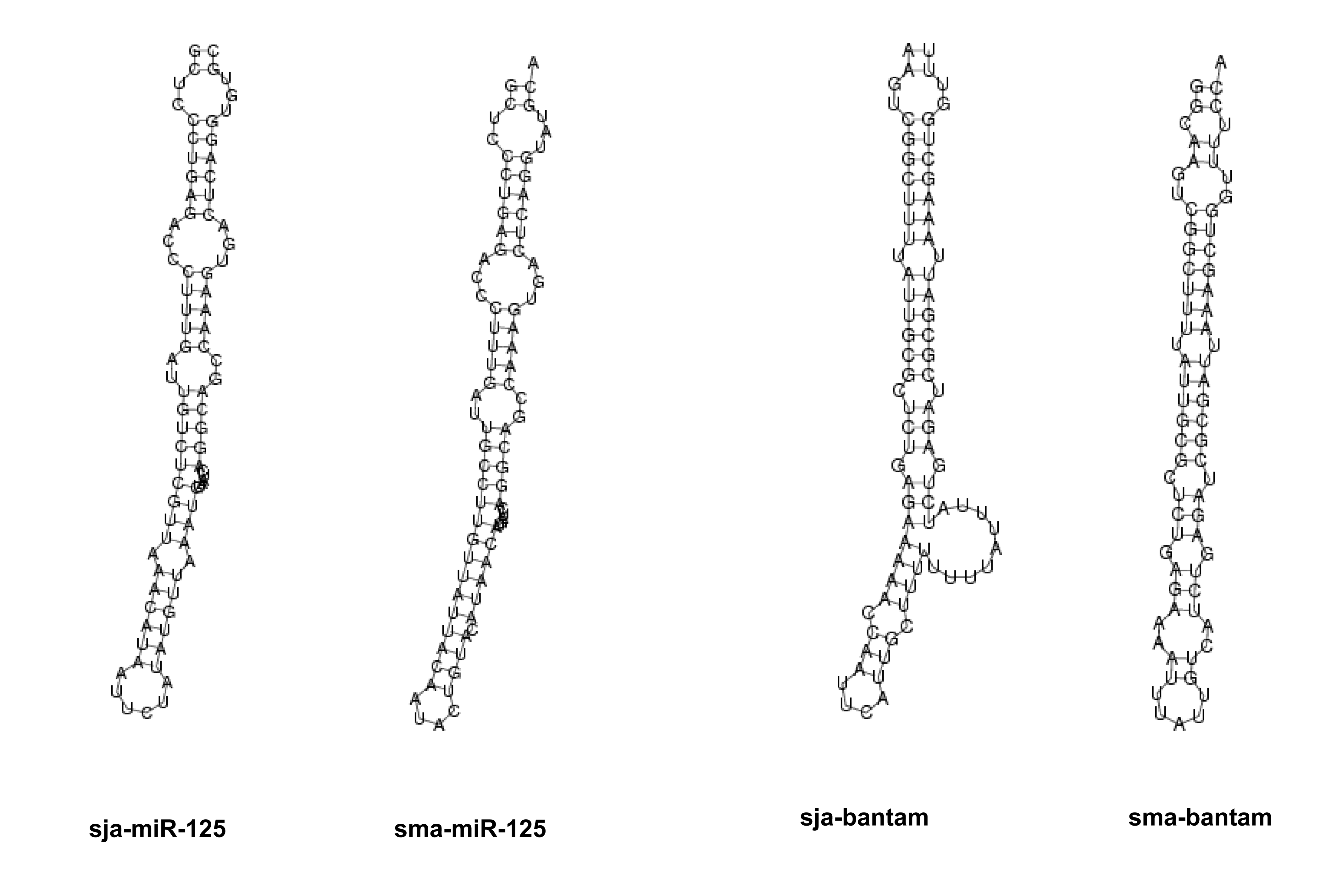

Supplement: Figure S1 — Predicted stem-loop structures for the mir-bantam and mir-125 precursors of S. japonicum and S. mansoni using Vienna RNAfold method. Mir-125 precursor from both S. japonicum and S.mansoni genome showed acceptable bulges in their secondary structure by Vienna RNAfold method. The bugle in the secondary structure of bantam precursor from S.mansoni was disappeared while the bugle from S. japonicum genome remains. (0.92 MB TIF) [file pone.0004034.s003.tif]

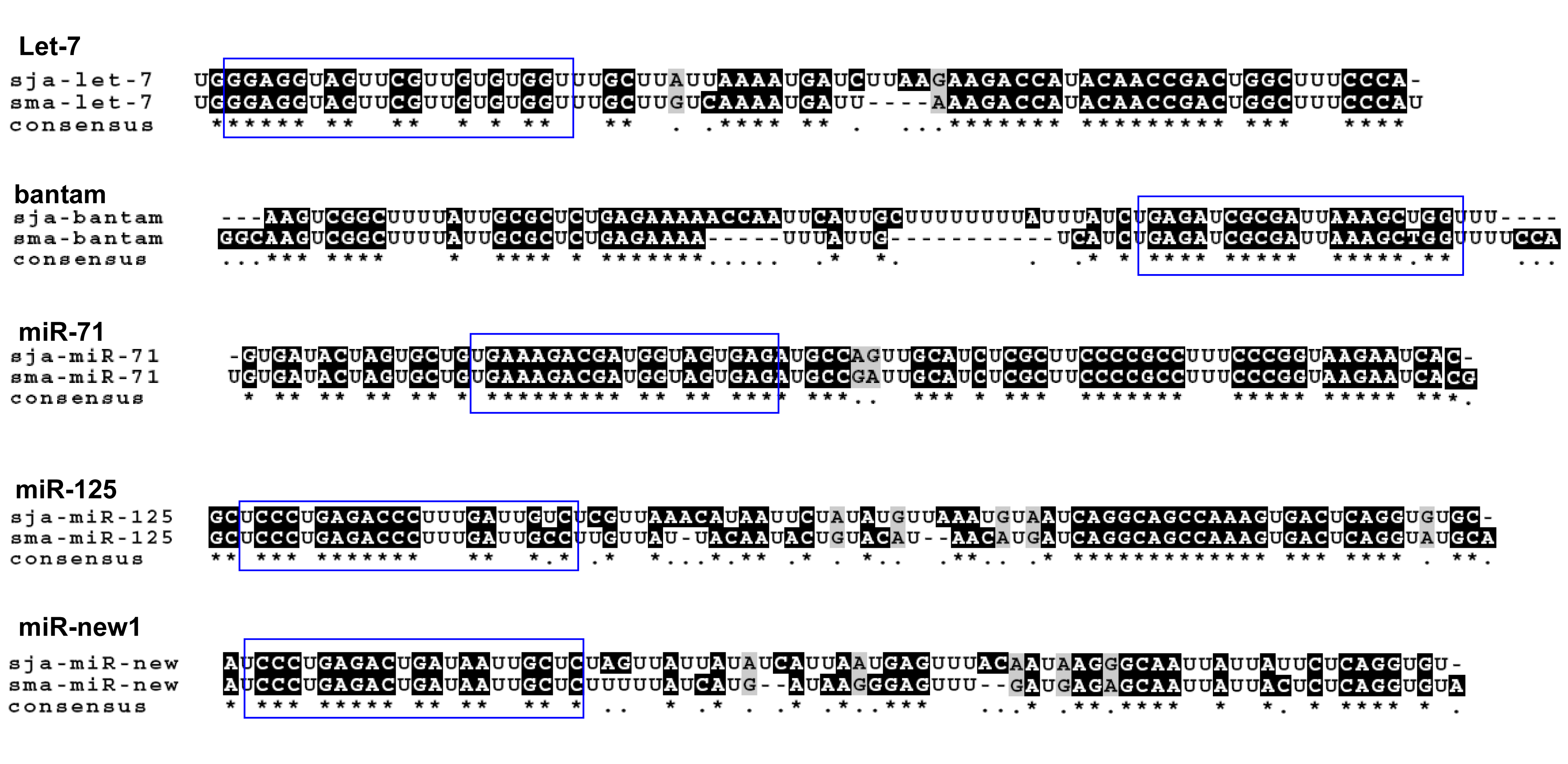

Supplement: Figure S2 — Alignments of S. japonicum miRNAs precursors with S.mansoni. The sequences of mature microRNAs are boxed. (1.19 MB TIF) [file pone.0004034.s004.tif]

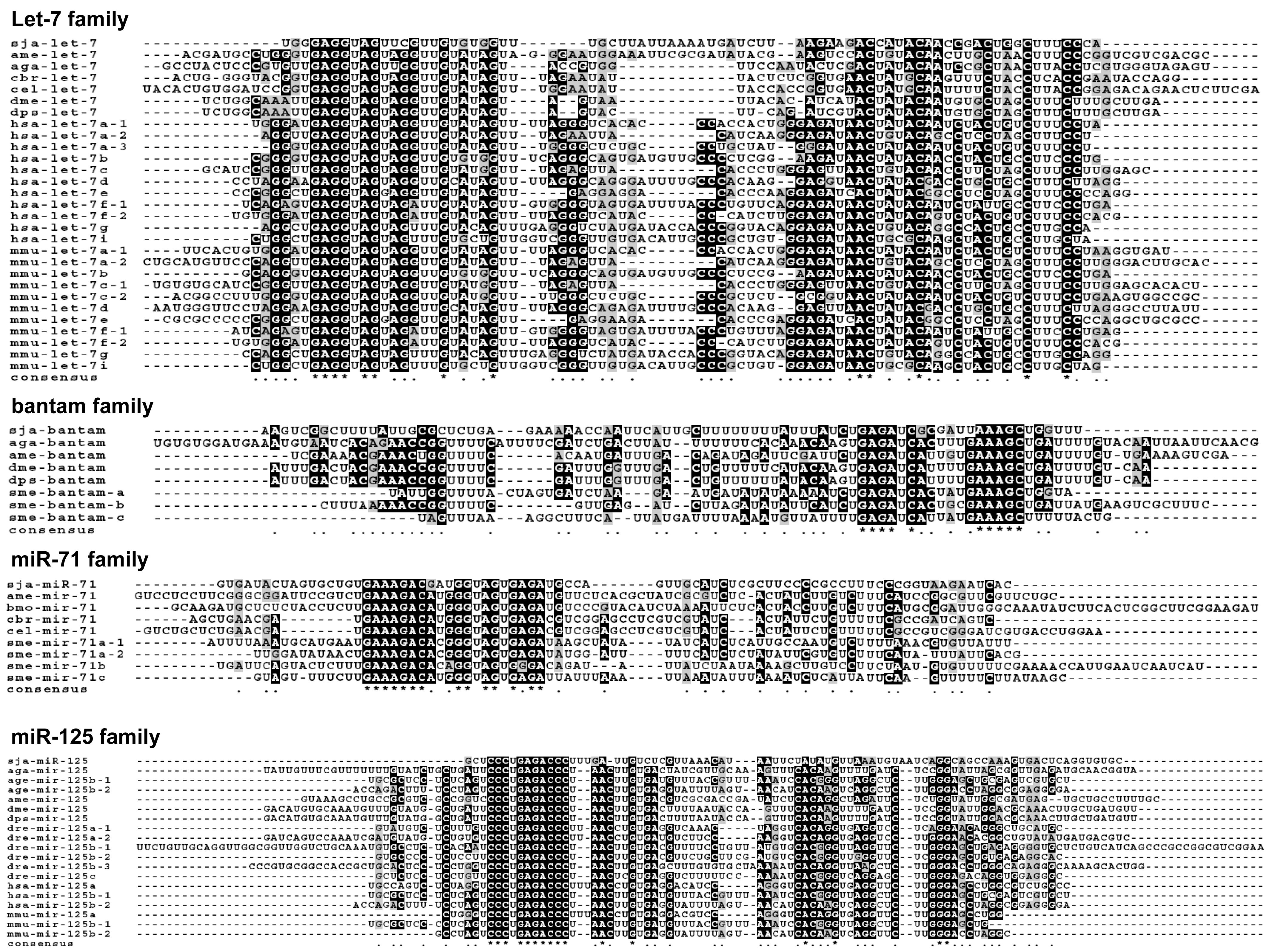

Supplement: Figure S3 — Sequence alignments of pre-miRNAs in each miRNA family. Abbreviation is the same as Figure 3. (4.50 MB TIF) [file pone.0004034.s005.tif]

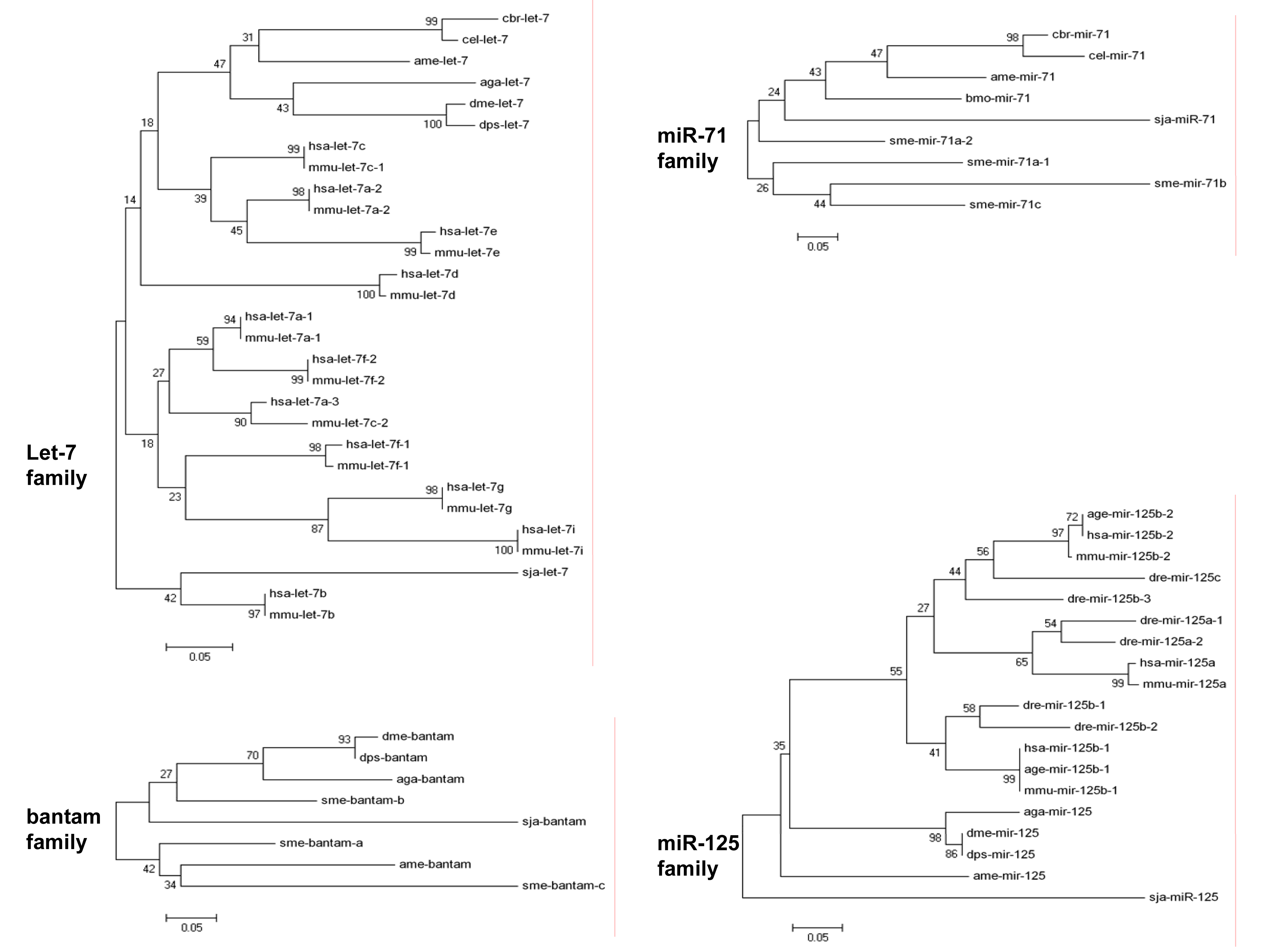

Supplement: Figure S4 — Phylogeny analysis of four miRNAs precursors. Abbreviation is the same as Figure 3. (0.74 MB TIF) [file pone.0004034.s006.tif]

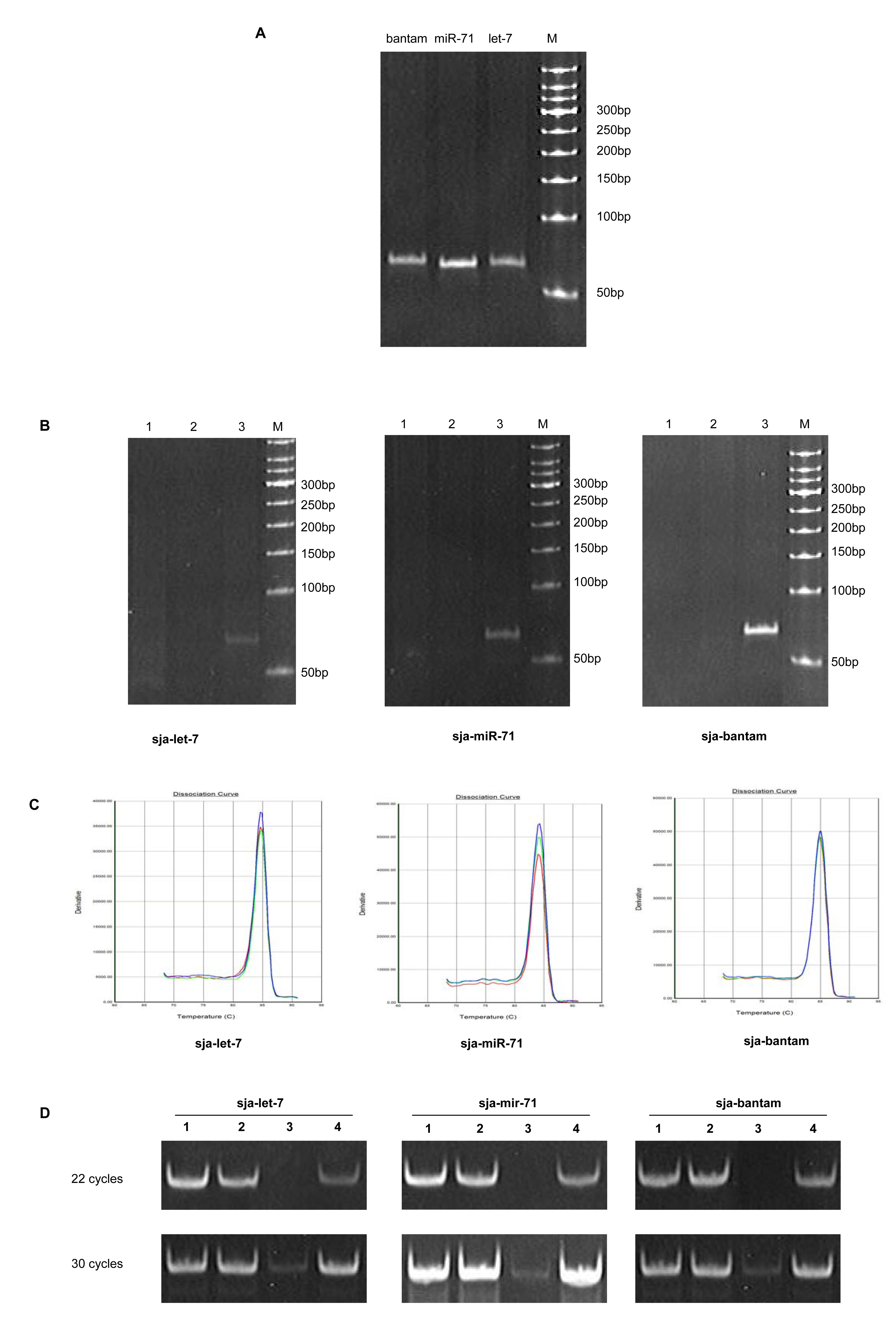

Supplement: Figure S5 — The specificity of stem-loop RT-PCR for S. japonicum miRNA quantitation. (A) Electrophoresis of the miRNA RT-PCR products on 8% PAGE showing bands of the anticipated size (65 bp). (B) Electrophoresis of the miRNA RT-PCR products on 8% PAGE showing no amplification using host RNA template. Lane 1: total RNA isolated from total blood cells of rabbit, lane 2: total RNA isolated from total blood cells of mice, lane 3: total RNA isolated from S. Japonicum, lane S: stem-loop RT-PCR reactions using the RNA of S. Japonicum adult worm with the mice mmu-let-7c specific primers. (C) Dissociation curves for the three duplicate RT-PCR reactions showing specificity of the reactions. (D) The specificity of the stem-loop RT-PCR assay in detecting mature miRNA expression. Four different RNA templates (lanes 1–4) were subjected to stem-loop RT PCR using the indicated stem-loop RT primer, then electrophoresed on 8% PAGE. Lane 1, 5 µg total RNA, lane 2, low molecular weight (LMW) RNA purified from 5 µg total RNA , lane 3, miRNA precursors (60–100 nt) isolated from 5 µg total RNA on denaturing 15% polyacrylamide gel, lane 4, mature miRNA (18–26 nt) isolated from 5 µg total RNA on denaturing 15% polyacrylamide gel. PCR cycle numbers are indicated to the left. (3.95 MB TIF) [file pone.0004034.s007.tif]

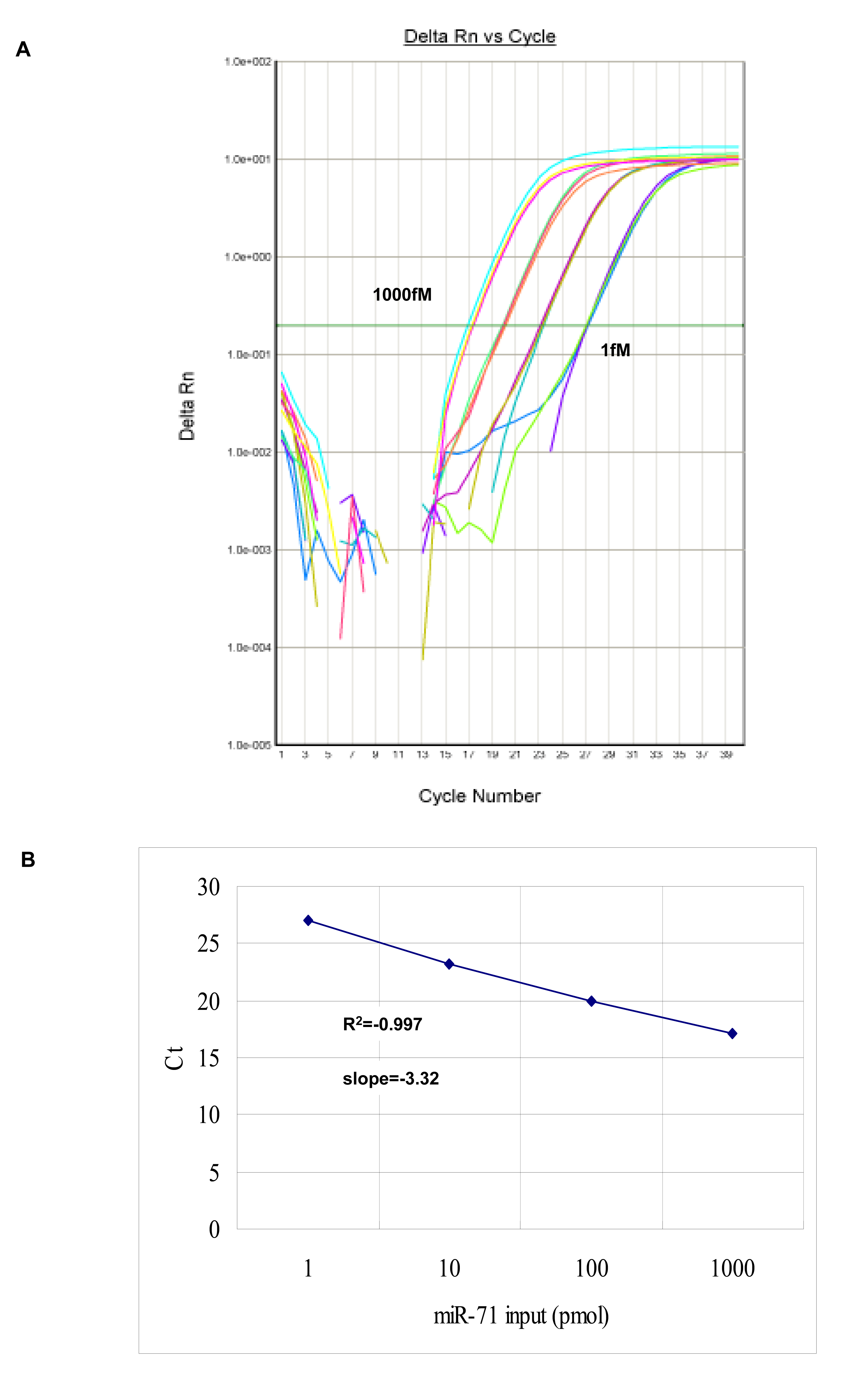

Supplement: Figure S6 — Dynamic range and sensitivity of sja-miR-71 RT-QPCR assay using synthetic sja-miR-71 miRNA. (A) Amplification plot of synthetic sja-miR-71 miRNA over three orders of magnitude. Synthetic RNA input ranged from 1pM to 1000pM in PCR. (B) Standard curve of the sja-miR-71 miRNA. (0.94 MB TIF) [file pone.0004034.s008.tif]

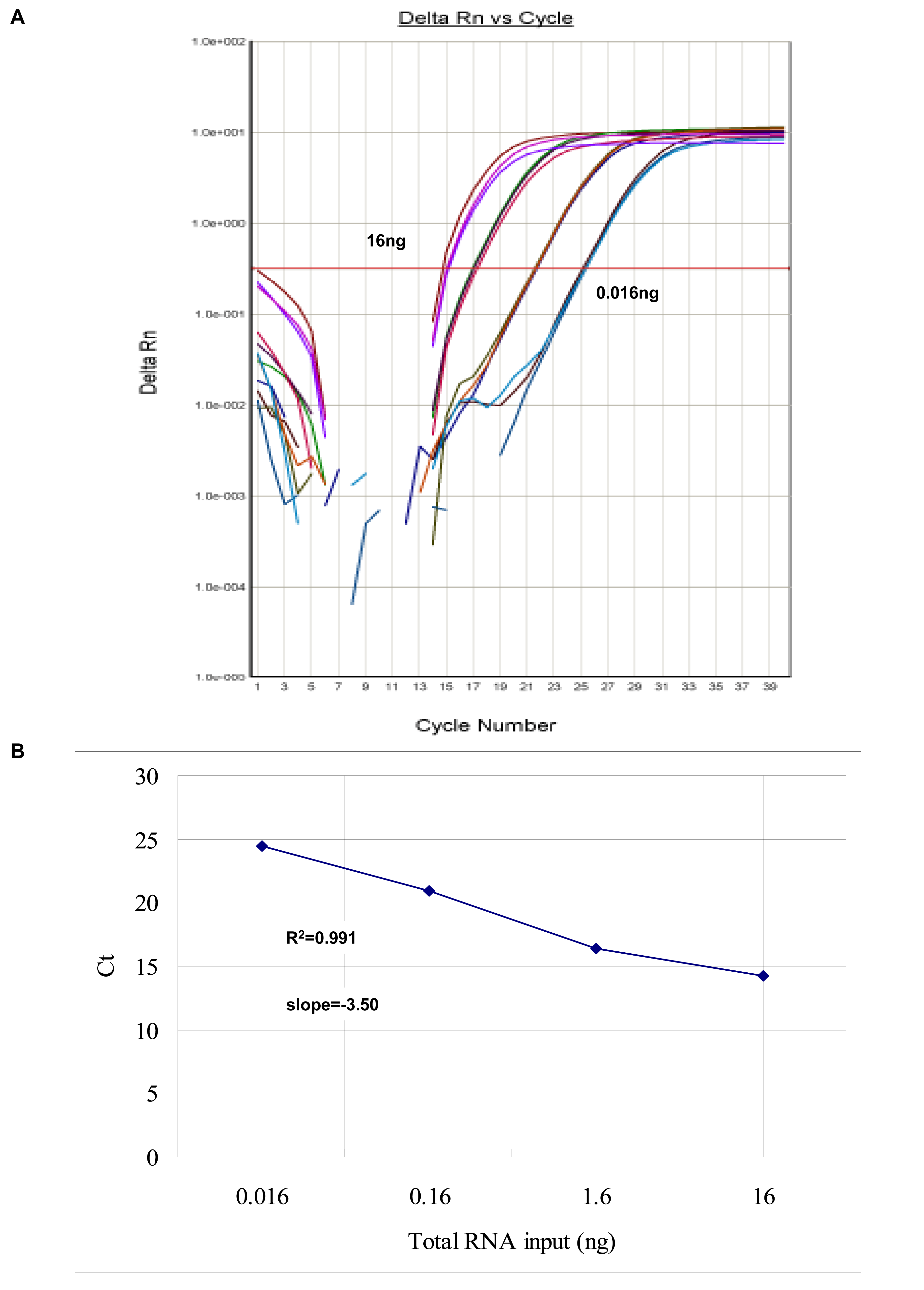

Supplement: Figure S7 — Dynamic range of sja-miR-71 RT-QPCR assay using adult S. japonicum total RNA. (A) Amplification plot of sja-miR-71 miRNA over three orders of magnitude. Total RNA input ranged from 0.016 to 16 ng per RT reaction. (B) Correlation of total RNA input to the threshold of cycle (CT) values of sja-miR-71 miRNA assays. (0.96 MB TIF) [file pone.0004034.s009.tif]

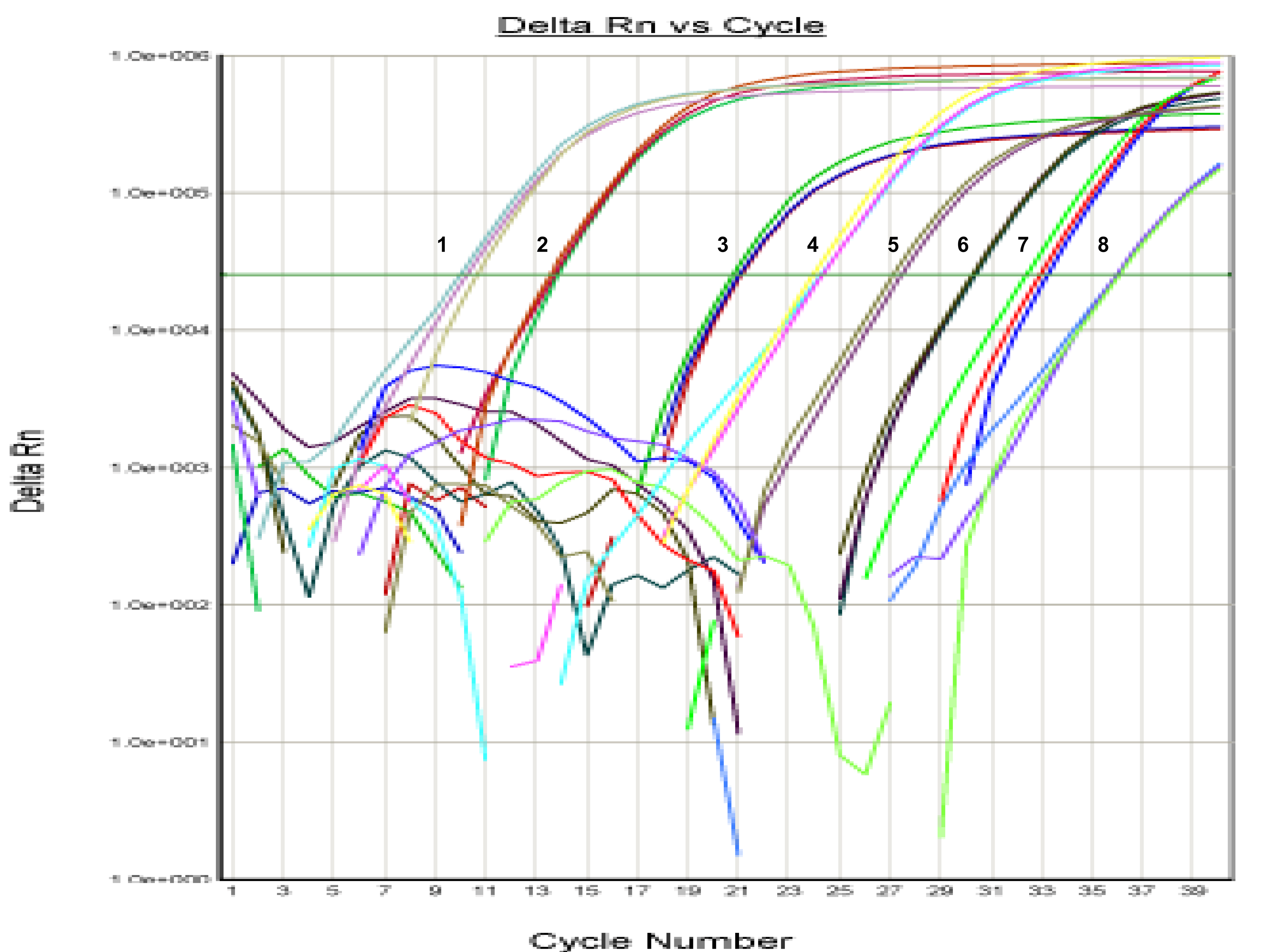

Supplement: Figure S8 — The amplification plot of sja-let-7, sja-mir-71, sja-bantam and alpha tubulin. The cDNAs for QPCR were obtained from the RT reactions that used the same cercaria sample. The RT reactions and consequent real-time quantitative PCR were performed at the same conditions. 1, 2, 3 and 4 were the amplification plot of sja-mir-71, sja-bantam, sja-let-7 and alpha tubulin, respectively, the RT product of cercaria RNAs. 5, 6, 7 and 8 were the amplification plot of sja-let-7, sja-mir-71, alpha tubulin and sja-bantam, respectively, using the no-RT control. (2.30 MB TIF) [file pone.0004034.s010.tif]
